# The long-term sustainability of a respiratory culture nudge

**DOI:** 10.1017/ash.2022.5

**Published:** 2022-01-31

**Authors:** Supreet Kaur, Mary Hutton, Rachel M. Kenney, Allison Weinmann, Linoj Samuel, Robert Tibbetts, Susan L. Davis, Corey Medler

**Affiliations:** 1 Department of Pharmacy Practice, Wayne State University Eugene Applebaum College of Pharmacy and Health Sciences, Detroit, Michigan; 2 Department of Pharmacy Services, Henry Ford Health System, Detroit, Michigan; 3 Department of Pharmacy, Intermountain Healthcare, Provo, Utah; 4 Office of Clinical Quality and Safety and Division of Infectious Diseases, Henry Ford Health System, Detroit, Michigan; 5 Department of Pathology and Laboratory Medicine, Henry Ford Health System, Detroit, Michigan

## Abstract

Resource-intensive interventions and education are susceptible to a lack of long-term sustainability and regression to the mean. The respiratory culture nudge changed reporting to “Commensal Respiratory Flora only: No *S. aureus*/MRSA or *P. aeruginosa.*” This study demonstrated sustained reduction in broad-spectrum antibiotic duration and long-term sustainability 3 years after implementation.

Simple and sustainable interventions are needed to improve antibiotic use. In 2016, an antimicrobial stewardship intervention by Musgrove et al^1^ was implemented that standardized reporting of microbiology reports to emphasize the absence of methicillin-resistant *Staphylococcus aureus* (MRSA) and *Pseudomonas aeruginosa* (PsA) in respiratory culture samples through a respiratory culture nudge.^1^ This intervention demonstrated improved antibiotic prescribing patterns and outcomes in patients empirically treated for pneumonia.^1^ However, many initially successful interventions fail to change long-term behavior, and the impact may diminish over time.^2^ Many factors can lead interventions to trail off, ranging from alert fatigue to failure to appreciate the impact on patient safety to the discontinuation of concentrated in-person refreshers or education.^2^ Thus, we evaluated the sustainability of the respiratory culture nudge on prescribing patterns and patient outcomes.

## Methods

We conducted a quasi-experimental study to determine sustainability of a microbiology nudge comment intervention at an 877-bed, tertiary-care, academic medical center in Detroit, Michigan. We assigned 2 comparator groups for this study. The first group was the early postintervention group (referred to as the early group), with patients admitted between August 1, 2016 and January 31, 2017. This intervention included the respiratory culture nudge comment and educational initiatives: in-person education and distributing educational handouts to the intensive care unit prescribers and inpatient pharmacists at department meetings. The second group was the long-term follow-up group (referred to as the late group), with patients admitted between August 1, 2018, and January 31, 2019. The late group maintained the nudge; however, no additional education was completed.

The original intervention occurred in 2016, when the microbiology laboratory report for commensal respiratory flora was modified from “commensal respiratory flora” to “Commensal Respiratory Flora only: No *S. aureus*/MRSA or *P. aeruginosa*,” when respiratory cultures lack significant growth quantities to require further work under standard guidelines.^1^ This comment serves as a prompt for teams to de-escalate antimicrobial coverage, specifically targeting the reduction of MRSA and PsA coverage. Other antimicrobial prescribing processes follow the usual standard of care for antimicrobial stewardship according to institutional guidelines. Patients met inclusion criteria if they were aged >18 years with a respiratory culture result for “Commensal Respiratory Flora only: No *S. aureus*/MRSA *or P. aeruginosa*” while receiving empiric antibiotic therapy for PsA and/or MRSA for a respiratory tract infection. The study was approved by the hospital institutional review board.

## Results

In total, 316 patients were included in this postintervention study: 105 in the early group and 211 in the late group. There were no significant differences in demographics (Table 1). All patients in the early group received empiric therapy targeting both MRSA and PsA compared to 191 (90.5%) of 211 patients receiving MRSA coverage and 186 (88.2%) of 211 receiving PsA coverage in the late group. MRSA antibiotics were de-escalated for 75 (71.4%) of 105 patients in the early group and 161 (84.3%) of 191 patients in the late group (odds ratio [OR], 2.15; 95% confidence interval [CI], 1.21–3.82; *P* < .008). For patients with MRSA de-escalation, the early group de-escalated within 24 hours of the result for 38 (50.7%) of 75 patients, with the late group de-escalating 114 (70.8%) of 161 patients (OR, 2.36; 95% CI, 1.34–4.16; *P* = .003). PsA antibiotics were de-escalated in 74 (70.5%) of 105 patients in the early group and 131 (75.8%) of 186 patients in the late group (OR, 1.31; 95% CI, 0.77–2.25; *P* < .320).

**Table 1.** Patient Characteristics and Outcomes

Characteristic or Outcome	Early Group (N = 105), No. (%)^a^	Late Group (N = 211), No. (%)^a^	*P* Value
Age, median y	61	64	.348
Sex, male	59 (56)	120 (57)	.908
ICU admission	70 (67)	118 (56)	.067
WBC > 12 or <4	50 (48)	72 (34)	.020
Received vasopressors	11 (11)	60 (28)	<.001
Ventilated	28 (27)	97 (46)	.001
Pneumonia diagnosed >48 h after hospitalization	19 (18)	42 (20)	.701
Immunocompromised	25 (24)	34 (16)	.098
LOS, median d (IQR)	10 [6–15]	8 [5–14]	.154
ICU LOS, median d	8 [4–15]	5 [3–11]	.029
MRSA DOT, median d (IQR)	5 [4–7]	3 [2–5]	<.001
PsA DOT, median d (IQR)	5 [4–7]	4 [3–5]	<.001
Charlson comorbidity score, median (IQR)	2 [1–4]	3 [1–5]	.091

Note. ICU, intensive care unit; WBC, white blood cell count; LOS, length of stay; IQR, interquartile range; MRSA, methicillin-resistant *Staphylococcus aureus*; DOT, days of therapy.

a Units unless otherwise noted.

Regarding patients with PsA de-escalation, the early group de-escalated therapy within 24 hours for 36 (48.6%) of 74 patients with the late group de-escalating 96 (68.1%) of 141 patients (OR, 2.25; 95% CI, 1.26–4.01; *P* = .005). Overall, 182 (86.3%) of 211 patients in the late group had MRSA or PsA antibiotic de-escalation or discontinuation after the nudge comment compared to 77 (73.3%) of 105 patients in the early group (OR, 2.28; 95% CI, 1.27–4.09; *P* < .005).

Antibiotic duration for MRSA coverage continued for a median duration of 5 days (interquartile range [IQR], 4–7 days) in the early group compared to median of 3 days (IQR, 2–5 days) in the late group (*P* < .001). Pseudomonal antibiotic coverage continued for a median duration of 5 days (IQR, 4–7 days) in the early group and median of 4 days (IQR, 3–5) in the late group (*P* < .001).

The most common antibiotic associated adverse event was acute kidney injury (AKI). AKI occurred in 15 (14.3%) of 105 patients in the early group compared to 13 (6.2%) of 211 patients in the late group (OR, 0.394; 95% CI, 0.18–0.86; *P* = .17). Patients receiving concurrent nephrotoxic agents occurred in 73 (69.5%) of 105 patients in the early group and 89 (46.4%) of 211 patients in the late group (OR, 0.38; 95% CI, 0.23–0.62; *P* < .001). There was no difference in *Clostridioides difficile* infection.

## Discussion

Three years after implementing the microbiology laboratory respiratory culture nudge, the intervention has continued to be associated with reduction of broad-spectrum coverage and decrease in antibiotic associated adverse events. Antibiotic duration for pseudomonal and MRSA coverage was reduced immediately after the intervention and continued to reduce in the late group. This respiratory nudge previously demonstrated reduced antibiotic harm,^1^ most notably AKI, which was sustained.

Previous studies have shown a concern for long-term sustainability of resource-intensive interventions. A study by Meeker et al^3^utilized a behavioral antibiotic stewardship intervention that reduced the rate of inappropriate antibiotic use for respiratory tract infections within a primary care practice. However, a follow-up was performed 12 months after stopping the intervention which showed an increase in inappropriate antibiotic prescribing and concern for long-term sustainability.^4^ Additionally, a study by Gerber et al^5^ used an audit and feedback intervention that improved adherence to prescribing guidelines. In a follow-up study by Gerber et al,^6^ the prescribing practice benefit was lost after the removal of audit and feedback intervention.

This study had several limitations. The Infectious Diseases Society of America (IDSA) released guidelines on the management of hospital-acquired pneumonia (HAP) and ventilator-associated pneumonia (VAP) in 2016.^6^ Although these guidelines may have contributed to maturation in overall pneumonia management, institutional risk factors for empiric MRSA and PsA coverage were consistent throughout the study. In July 2018, a pharmacist-driven MRSA nares intervention was implemented to assist with reducing unnecessary MRSA coverage. This intervention may have influenced the discontinuation of MRSA coverage; however, no new intervention targeting antipseudomonal therapy was undertaken.

Education and other labor-intensive interventions focused on changing antibiotic prescribing are notoriously short-lived due to a variety of challenges and barriers to long-term success. Challenges and barriers include sustainability, competing priorities, and a tendency for behavior to regress to the mean after initial successes. However, in contrast to the other antibiotic stewardship interventions, the commensal respiratory flora laboratory reporting comment, which is automated, has resulted in sustained improvement long after implementation. At the time of implementation, the stewardship team did perform widespread education throughout our health care system. However, sustained success has occurred with no additional education or stewardship intervention beyond the standard of care. We attribute this result to the broad multidisciplinary approach in designing the intervention, the simplicity, directness, and timeliness of the respiratory comment, and the prioritization of antimicrobial stewardship infrastructure and support.
